# Laminin N-terminus (LaNt) proteins, laminins and basement membrane regulation

**DOI:** 10.1042/BST20210240

**Published:** 2022-11-10

**Authors:** Natasha D. Chavda, Bilge Sari, Fawziah M. Asiri, Kevin J. Hamill

**Affiliations:** Institute of Life Course and Medical Sciences, University of Liverpool, 6 West Derby Street, Liverpool L78TX, U.K.

**Keywords:** basement membrane, laminin, netrin

## Abstract

Basement membranes (BMs) are structured regions of the extracellular matrix that provide multiple functions including physical support and acting as a barrier, as a repository for nutrients and growth factors, and as biophysical signalling hubs. At the core of all BMs is the laminin (LM) family of proteins. These large heterotrimeric glycoproteins are essential for tissue integrity, and differences between LM family members represent a key nexus in dictating context and tissue-specific functions. These variations reflect genetic diversity within the family, which allows for multiple structurally and functionally distinct heterotrimers to be produced, each with different architectures and affinities for other matrix proteins and cell surface receptors. The ratios of these LM isoforms also influence the biophysical properties of a BM owing to differences in their relative ability to form polymers or networks. Intriguingly, the LM superfamily is further diversified through the related netrin family of proteins and through alternative splicing leading to the generation of non-LM short proteins known as the laminin N-terminus (LaNt) domain proteins. Both the netrins and LaNt proteins contain structural domains involved in LM-to-LM interaction and network assembly. Emerging findings indicate that one netrin and at least one LaNt protein can potently influence the structure and function of BMs, disrupting the networks, changing physical properties, and thereby influencing tissue function. These findings are altering the way that we think about LM polymerisation and, in the case of the LaNt proteins, suggest a hitherto unappreciated form of LM self-regulation.

## Introduction

Basement membranes (BMs) are specialised extracellular matrix (ECM) structures, underlying all epithelium, mesothelium and endothelium [1]. BMs provide physical support for cell attachment, act as semi-permeable barriers and influence cell behaviour through; direct signalling via interaction with cell surface receptors or through presenting tuneable biophysical interaction sites, indirectly influencing signalling via the sequestration and controlled release of signalling molecules. These overlapping but diverse functions mean that BMs are key modulators of a myriad of cell and tissue behaviours; these include: regulating cell migration during development, tissue remodelling and wound repair, controlling cellular movement during immune extravasation and tumour progression, and, via their signalling roles, defining differentiation and lineage specification [1–3]. Together these amount to critical functions and make BMs essential for multicellular life. Indeed, inherited or acquired disorders leading to dysfunction of individual BM components are often embryonic lethal or cause devastating human diseases, for example, major kidney problems in Alport syndrome and Pearson's syndrome, skin and other epithelial fragility in junctional epidermolysis bullosa, and severe muscular dystrophy in merosin-deficient congenital muscular dystrophy, as reviewed in [4–7].

At the core of every BM are two networks of proteins: a collagen IV network and a laminin (LM) network. These are connected via nidogens and heparan sulfate chains of the proteoglycans perlecan and agrin [3,8–11]. There are six different α-chain subunits of collagen IV, which form different heterotrimers, with α1α1α2 being the most common [1]. However, the LM family provides greater flexibility for tissue specialisation and is described in more detail below [2].

One of the most intriguing aspects of BM biology are the localised differences in structure and function. Whereas, historically, BM studies relied on electron microscopy approaches on dehydrated tissue; more recently atomic force microscopy (AFM), mass spectrometry and advanced light microscopy imaging have revealed complexities in biophysical properties including stiffness, in BM structure and protein composition that differ depending on the tissue. Indeed, BMs are no longer considered as static physical structures but rather undergo remodelling throughout life via the action of proteolytic processing and non-disruptive network modifying proteins [12,13], and/or through contextual differences in protein expression. Human and mouse studies focused on the inner limiting membrane (ILM) of the retina, Descemet's membrane supporting the corneal endothelium, lens capsule and retinal blood vessels have revealed a general trend that with ageing, BMs become thicker, more amorphous and change biochemical composition with the relative concentrations of collagen IV and agrin increased and LMs reduced [14–19].

AFM studies have revealed a distinct sided-ness to BMs, with the LM-enriched epithelial/endothelial side being considerably stiffer than the stromal-facing aspect [20], and ILM stiffness greater on the retinal side than the vitreous side [19,21]. Stochastic optical reconstruction microscopy (STORM) microscopy has identified that the glomerular BM, which also thickens with age, to be highly structured and laminar, not amorphous as previously thought [22,23]. Moreover, localised differences in biophysics have been established in drosophila egg elongation studies, where AFM measurements showed BM stiffness anisotropy between the centre and posterior regions. Within this context, collagen IV was central to defining the bulk properties of the BM whereas the LM mechanical contribution was more nuanced, with LM knockdown eggs showing increased stiffness in the centre region but decreased stiffness in the posterior region in comparison with controls [21]. These recent advances in appreciation of the biomechanical and structural features of BM places greater emphasis on understanding how the core proteins of LMs self-assemble to form the different types of BM.

## Laminins

Each LM is a glycoprotein heterotrimer composed of three subunits: an α-, a β- and a γ-chain, with each chain derived from a distinct gene. In humans, there are five α-chain (LAMA1–5), three β-chain (LAMB1–3) and three γ-chain (LAMC1–3) genes [2,14]. A fourth β has been identified; however, this has not been found in any heterotrimers and may be a pseudogene. Additionally, LAMA3 contains two distinct promoters which generate functionally and structurally distinct subunits; a ‘full-length' form, LMα3B and a much shorter LMα3A [15,16]. The naming convention for the assembled LM heterotrimers reflects the subunit composition with the LM formed from α1, β1 and γ1 subunits being known as LM111 [17].

**Figure 1.**
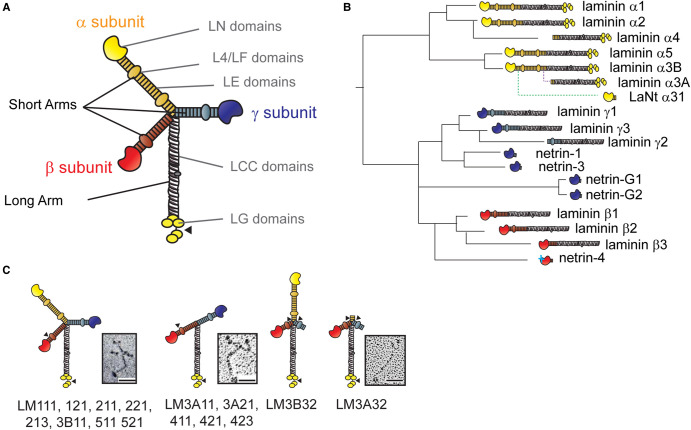
Laminin domain architecture, evolution and heterotrimer assemblies. (**A**) Archetypal laminin structure. Yellow, red and blue regions indicate short arms generated from individual laminin chains, grey represents the long arm. LN — laminin N-terminus domain, LE — laminin-type epidermal growth factor-like domain, L4/LF — laminin domain IV globular domain, LCC — laminin coiled-coil domain, LG — laminin globular domain. (**B**) Derivation, relatedness and approximated order of appearance of laminin family members. Solid lines represent gene duplication and rearrangement events. Dotted lines indicate the evolution of an additional promoter (for LMα3A) and of intron-retention and alternative polyadenylation for LaNt α31. (**C**) Diagram of different laminin assemblies. Numbers represent chain composition e.g. LM111 is laminin α1β1γ1. Arrowheads indicate sites of proteolytic processing. Inset images are rotary shadowing electron microscopy images of purified LM111 (left) from [55], LM411 (middle) from [43] and LM332 (right) from [30]. Scale bars 50 mm. These images have been cropped from the original figures and are re-used in accordance with Creative Commons BY 4.0 license agreement.

The archetypal LM heterotrimer structure is cross-shaped [24], formed of one long arm and three short arms (Figure 1A). The long arms comprise the defining feature of LMs; a 561-591 amino acid LM coiled-coil (LCC) domain through which the chains combine into an α-helical coiled-coil with disulfide bonds stabilising the structure [2]. The formation of the coiled-coil is dependent upon critical residues toward the C-terminal regions of the LCC, which means not all αβγ combinations are possible, with 16 having been purified in mammals [25–28]. In the α-chains, five LM globular (LG) domains are attached C-terminally to the LCC. These comprise a LG1-3 trio and a LG4-5 duo connected to one another by a linker region/hinge. The LG domains contain most of the high-affinity binding sites for LM cell surface receptors, including integrins, syndecans and α-dystroglycan [2,3]. Proteolytic processing can occur within the linker region, releasing the LG4-5 domain [29] and having implications for how cells interact with the LMs, and is associated, for example, with hemidesmosome maturation [30-32]. Indeed, like most multi-domain ECM proteins, multiple proteolytic processing events can potentially unmask matricryptic sites [33-36]. The LG domains also appear to contribute to the ability of BMs to sequester and control the release of growth factors, with vascular endothelial cell growth factor, platelet-derived growth factor, fibroblast growth factor, bone morphogenetic protein and neurotrophin families all capable of binding to LMs via the heparan-binding domains located on α-chain LG domains [37,38].

The LM short arms are each comprised of the amino-terminal end of one individual chain [39,40]. At the end of most short arms are LM N-terminal (LN) globular domains, of 228–259 residues. These are node-forming globules involved in LM self-polymerisation and network formation, described below [3]. The majority of the short arms are formed of rod-like arrays of LE domains, which, with the LN domain, give a distinct ‘stalk and flowerhead' structure [41]. Each LE domain contains eight cysteine residues, paired together by disulfide bonds, forming interconnected loops [42]. In chains apart from LMα3A, α4 and β3, these LE arrays are interrupted by either an L4 domain inserted between residues three and four of one LE domain or an LF domain located between LE arrays [2,42]. Gene duplication and domain rearrangements have meant that not all the LM chains contain all structural domains (Figure 1B). Of particularly relevance when considering LM polymerisation is the existence of three chains that have much shorter N-terminal arms (LMα3a, α4 and γ2), with heterotrimers containing these chains taking on either a T-shape (LM3A11, 3A21, 411, 421, 423) [43] or more rod-like shape (LM3B32 and 3A32) [30,44] (Figure 1C).

The presence and distribution of the different LM types are tissue-specific and changes during development and tissue remodelling. In generalised terms, LM111 is ubiquitous during embryogenesis, but becomes tissue-specific in the adult as α5 containing LMs become more common [45,46]. As development continues, different LM heterotrimers become associated with certain tissue types, for example, LM332 is enriched in most epithelial basal lamina, LM411 and LM421 throughout the vasculature endothelium and LM211 and LM221 in muscle BMs [1–3,47–49]. However, most tissues contain a combination of different LMs that overlap or can be spatially or temporally distinct. These localised differences can be functionally important; for example, both LM511 and LM332 are abundant in developing and adult skin [50]; however, in the hair cycle LM511 induces hair growth with a steady increase in expression during anagen, while LM332 has a suppressing effect on hair growth and is down-regulated. Conversely, during catagen, LM332 is up-regulated, while LM511 is down-regulated [51,52]. BM LM composition changes with age are seen with decreased LM511 due to photoageing in epidermal BM [53], with differences also observed between infants and adult human corneal BMs [54].

## LM network formation

**Figure 2.**
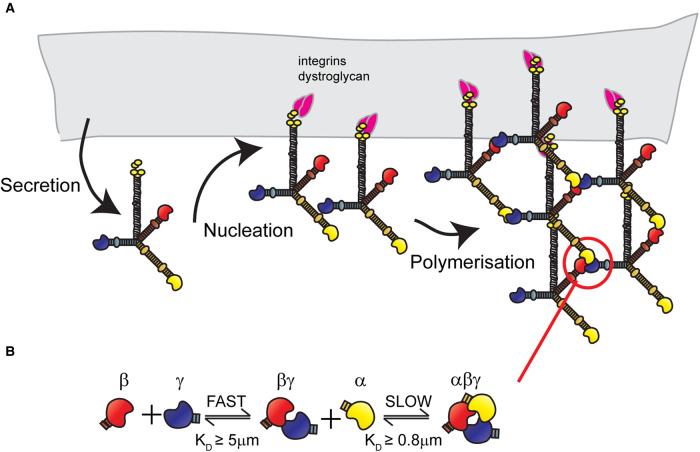
Laminin network assembly. (**A**) Network assembly involves, first, secretion of the fully trimerised laminin protein. Then, in the ‘nucleation' step, the proteins bind to cell surfaces receptors including integrins α3β1, α6β1 and α6β4 or dystroglycans. Once sufficient local concentration is achieved through receptor clustering, the ‘polymerisation' step occurs via interaction between laminin N-terminal domains. (**B**) Laminin ternary node formation is a two-step process involving a rapid formation of relatively unstable βγ dimers, followed by slower interaction with an α laminin N-terminal domain to form a more stable αβγ ternary node.

LM network self-assembly is LN domain dependant and occurs via a two-step process of nucleation, where the LMs bind to cell surface receptors, followed by propagation involving LN domain interaction [55,56] (Figure 2A). The network assembly steps can be further broken into a temperature-dependent oligomerisation step followed by a calcium-dependent polymerisation step [56]. For *in vitro* network assembly, all three short arms must contain a LN domain; leading to a ‘three-arm hypothesis’ [56,57]. Indeed, the arms must also be different, as the α, β and γ LN domains each play distinct roles [56-60] (Figure 2B). First, a fast but unstable interaction occurs between the β and γ LN domains. Then the βLN-γLN binary complex allows for a second, slower, stabilisation step to occur, involving an α LN binding to form an αβγ ternary node [61] (Figure 2B).

The LN domain crystal structures have shed further light on the polymerisation process [41,61]. Each LN domain is centred around a β-sandwich jelly-roll motif with differing loop regions between the chains. While the surfaces are not conserved between the α, β and γ families, there is conservation within each chain family of β-sandwich residues on one face and of shielding glycans on the other [41]. The exposed residues on the non-glycosylated faces of the β and γ LN domains mediate binding with the α LN domain [41,61]. Whereas the α LN domains contain a conserved surface loop, involved in the stabilisation of the βγ dimer. Recent elegant studies have further established that the LN domains interact in a heel-to-toe fashion, forming triskelion-like structures [59] (Figure 2B). These new findings imply that two distinct interaction sites exist between each LN domain, and that functional conservation of both is required for the stabilisation of the network [59]. This becomes particularly important when considering netrin-4.

## Netrins — widening the laminin superfamily

Phylogenetic analyses indicate that LM α-, β- and γ-chains and netrins were all generated from a shared common ancestor (Figure 1B). An early evolutionary duplication separated the LM α subunit ancestors from the β and γ subunits, then a second split occurred between the β and γ genes. Thereafter, the netrins evolved separately at least twice and possibly three times; generating netrins-1 and -3 from the γ-chain ancestor, netrin-G1 and -G2 from the βγ ancestor, and netrin-4 from the β-chain ancestor [62,63] (Figure 1B). The netrins lost the majority of the C-terminal LM domain, including the LCC domain, meaning netrins cannot form trimers. However, they contain LN domains and short stretches of LE repeats followed by a netrin-specific C-terminal domain [64].

The γ LM-like netrins are considered primarily signalling proteins with important roles in axonal guidance; acting via cell surface receptors including deleted in colorectal cancer (DCC), neogenin and UNC5 [65]. Netrin-1 is known to gather and guide axons in the central nervous system, but has a dual role in neural migration acting as a chemorepellent for oligodendrocyte precursors and parallel fibres, but a chemoattractant for dopaminergic and pre-cerebellar neurons [66-68]*.* Netrin-3 is also involved in axonal guidance but is less effective in signalling than netrin-1; it has been suggested that netrin-3's weak affinity for DCC may be the reason for its relatively lower effectiveness [69]. Intriguingly, growth-cone attraction toward netrin-1 has been shown to be converted to repulsion when the neurons are plated on LM111, suggesting interplay between these protein families [70].

Netrin-4, the only LM β-type netrin, is different from the rest of the family. Although it also promotes axon outgrowth [71], it is mostly known as a regulator of angiogenesis. Specifically, netrin-4 localises to blood vessels and improves angiogenesis in the peri-infarct cortex after focal cerebral ischaemia [72], and absence of netrin-4 is associated with alteration of vascular structure and spontaneous leakage in the retina [73]. From a mechanistic perspective, netrin-4's influence upon angiogenesis requires Unc5B and neogenin as when either are silenced, the inhibitory effect of overexpressed netrin-4 are stopped and a direct effect is suggested as both proteins co-immunoprecipitate with netrin-4 [74]. However, although these data imply direct signalling, it has now also been established that netrin-4 is a potent modulator of LM network assembly and contributes to defining BM biophysical characteristics.

**Figure 3.**
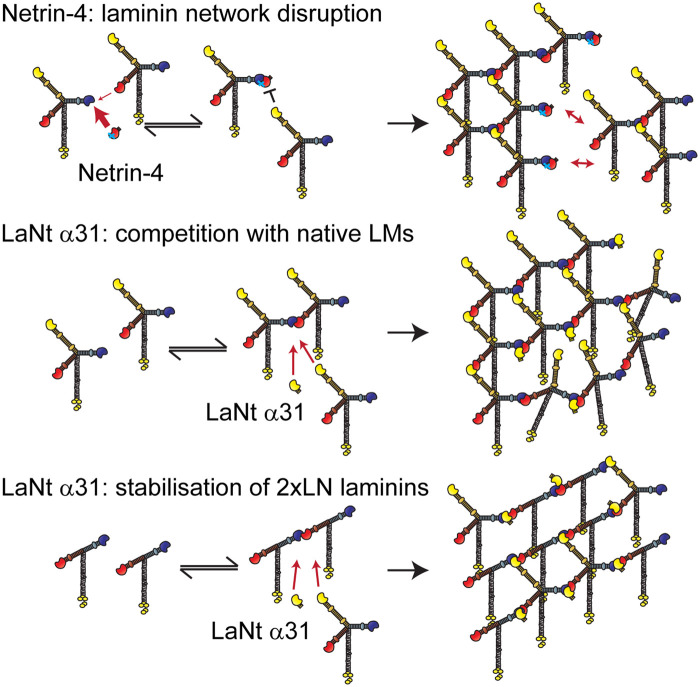
Netrin-4 and LaNt α31 predicted effects on laminin networks. (Top) Netrin-4 binding with high affinity to laminin γ-chains can outcompete the laminin β-chains. However, α-chains cannot bind to γ/netrin-4 complexes. Therefore, netrin-4 disrupts laminin networks leading to locally increased pore size and decreased basement membrane stiffness. (Middle) LaNt α31 has an identical laminin N-terminal domain as LMα3b and therefore LaNt α31 will compete with approximately equal affinity for α-chain binding sites. This will lead to a partially disrupted network with reduced stiffness, with the level of disruption proportional to the expression level. (Bottom) In basement membranes containing T-shaped laminins, where only the β- and γ-chains contain N-terminal domains (e.g. LM411), the LaNt α31 protein may stabilise transient βγ interactions allowing the formation of stable ternary nodes. This would lead to linear arrays of the two-arm laminins. These arrays could also be cross-linked via the integration of some three-arm laminins within the local structure. The mechanical properties of the formed network would depend on the ratio of the three-arm to two-arm laminins and the local LaNt α31 concentrations.

Central to the understanding of netrin-4 as a LM network disruptor is appreciating that although it contains a LM β-like LN domain, the domain itself is imperfect. The binding site for γ LN domains is conserved but the α LN binding site has been lost (Figure 3). Indeed, netrin-4 displays much higher affinity (2500× greater) than LMβ1 for LMγ1 LN domain [12]. In polymerisation assays, these differences mean that netrin-4 can not only prevent LM111 polymers from forming but can also disrupt preformed LM polymers through competing for the γ LN domain binding site [12,75], and this reduces the stiffness of LM containing hydrogels when netrin-4 is added as a recombinant protein [12]. In intact tissue, netrin-4 also may exhibit this non-enzymatic disruptive force on mature BM, with increased netrin-4 leading to larger pores and softening of BM mechanical properties [12,13]. These stiffness effects might be a key determinant of tumour invasion potential; softer tissues with higher netrin-4 to LM ratio display a resistance to metastasis formation [13]. This correlation between netrin-4 expression levels, tissue stiffness and tumour invasion does not preclude netrin-4 also acting through an alternative mechanism independently or in addition to its biophysical role.

## Laminin N-terminus proteins — the ‘missing' laminin superfamily members

While the netrins provide a source of β and γ type LN domains, alternative splicing from LM genes provides a means to produce α LN domain-containing protein fragments through a process of intron-retention and polyadenylation within the retained intron [76] (Figure 1B). At least one of these alternative splice isoforms from the LAMA3 gene has been confirmed at the protein level and is termed laminin N-terminus (LaNt) protein α31 (LaNt α31). The LaNt proteins are netrin-like in structure consisting of an α LN domain, LE repeats and a short unique C-terminus derived from the intronic sequence and could be considered the hitherto ‘missing' members of the LM/netrin family. Proteolytic processing of LM proteins can also lead to the release of LN domain-containing fragments and represent an additional mechanism to produce netrin-like proteins [60,77,78]. That multiple independent mechanisms exist to produce LN domain-containing proteins suggests important functional roles, and these are now beginning to become apparent.

**Figure 4.**
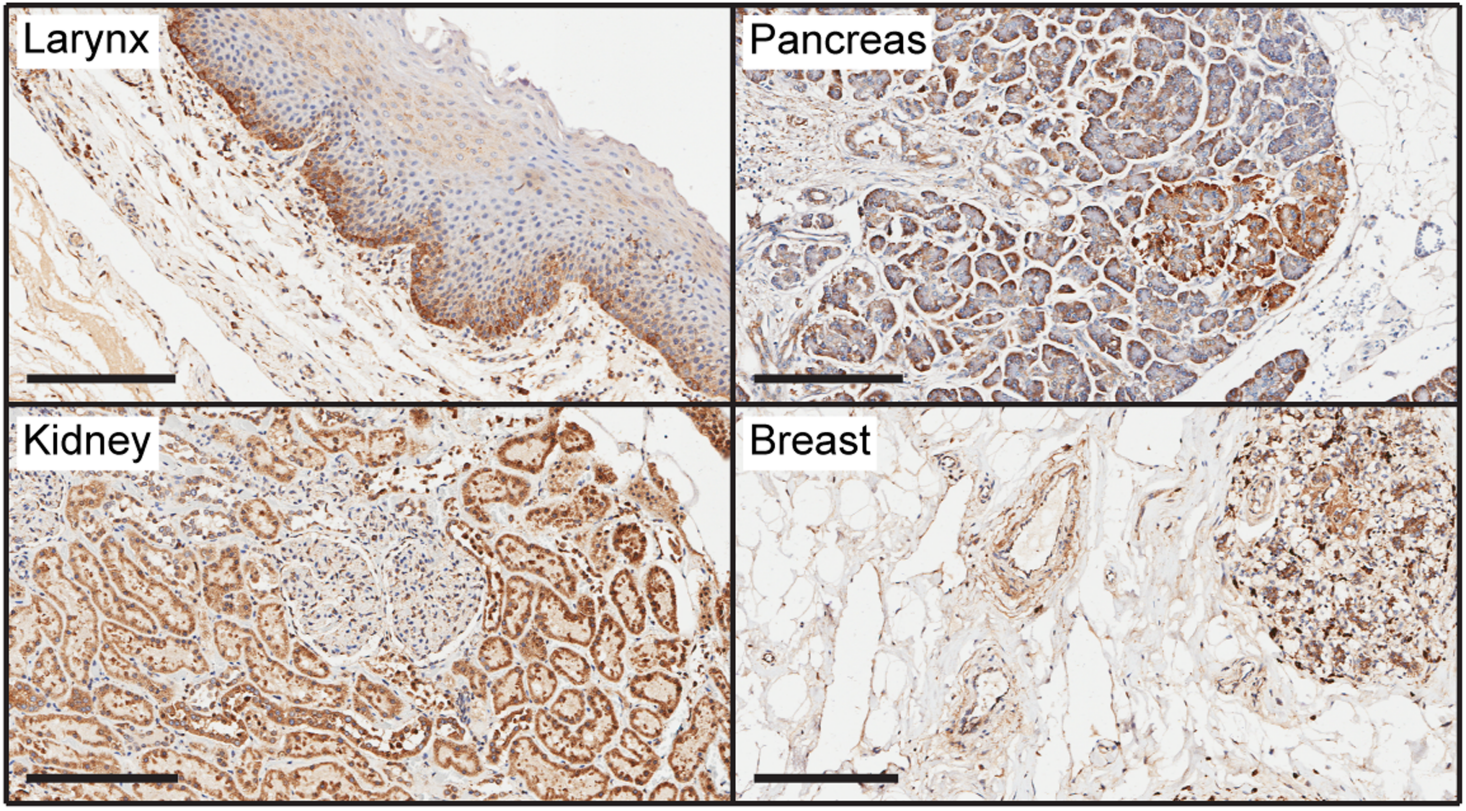
LaNt α31 distribution in human tissue. Immunohistochemistry images of formalin-fixed paraffin-embedded human larynx, pancreas, kidney and breast tissue sections obtain from US Biomax (MBN481, US Biomax, Rockville, Maryland, U.S.A.) processed with mouse monoclonal antibodies against LaNt α31 [79]. Scale bar 200 µm.

LaNt α31 displays widespread expression at the mRNA and protein level (Figure 4), including being enriched in the basal layer of most epithelium, throughout the vascular system, around terminal ducts in the breast, and in specialised cell types in the brain [79]. Increased expression has been identified in invasive breast ductal carcinoma, with further increases in distant metastases and with high expression associated with a non-cohesive tumour phenotype [80]. The expression also increased in 2D scratch wound assays during the early stages of *ex vivo* burn wound closure and in epithelial stem cell activation assays [76,81]. Functional studies have demonstrated that both down-regulation and increased expression of LaNt α31 lead to defects in epithelial cell adhesion with reduced migration rates associated with changes to LM332 organisation and hemidesmosome maturation [76,81,82]. In breast cancer cells, the increased expression changed invasive behaviour from coherent multicellular streaming to individual cell invasion. This effect was only observed for cells invading into LM containing hydrogels, suggesting a LM dependency of function [80]. *In vivo*, ectopic transgenic overexpression during the late stages of development led to non-viable offspring with neonates displaying extensive leaky blood vessels most likely attributable to defective vessel BMs [83].

The understanding of LM network formation and LN-LN interaction leads to a hypothesis where LaNt α31 could elicit its functional effects by competing with α LN domains of the LMs in a similar but distinct way to netrin-4. Unlike netrin-4's competition with β LN domains (Figure 3, Top), the LaNt proteins would compete with the α-chains during the stabilisation step of network assembly. Indeed, as LaNt LN domains are identical with the native LM, any competitive effect would be much weaker than netrin-4 at equivalent concentrations. Moreover, this LN domain conservation raises a very different potential situation. As they are perfect matches to the LMs, LaNt proteins could actually be integrated into the ternary node in the same way as the native LM α-chain. LaNt integration would still change the network structure and mechanics, but one could predict the effects to be subtler than the full localised disruption induced by netrin-4 (Figure 3, Middle). The perfect conservation and putative integration of the LaNt-βγ ternary node raises a further potential situation, where LaNt proteins could play a stabilising role in those BMs where the predominant LMs present are normally considered unable to self-assemble.

As LMα3A, α4- and γ2-chains all lack LN domains, any heterotrimers containing these chains are unable to independently polymerise under the ‘three-arm hypothesis' [56,57,78,84]; this includes LM311, LM321, LM411, LM421, LM423, all of which lack an α LN domain. A biological rationale for this scenario is that networks containing LMs with missing LN domains have different tissue biomechanical properties in terms of flexibility and stability than those with fully polymerised networks [44]. A good example of differential polymerisation status potential can be seen in blood vessels, where the 3xLN domain LM511 and 2xLN domain LM411 are the main LMs. These two LMs are found at different ratios dependent on the blood vessel type and their maturation state; lower expression of LM511 is observed in post-capillary venules relative to venules and relative to veins [85]. This leads distinct areas of defined LM411 to LM511 ratio. Intriguingly high LM511 sites have higher endothelial-to-endothelial cell junctional adhesion strength, and the low LM511 are preferred for leukocyte extravasation; although the reasons behind this later effect also reflect T-cell interactions with LM511 that inhibit migration [85-87].

The LaNt proteins raise a possibility where the additional free α LN domain allows for partial network assembly between non-network forming LMs, compensating for the missing α LN domain. This stabilisation effect would lead to a different assembly state; rather than robust hexagonal arrays that would be self-supporting, the 2xLN + LaNt nodes would be predicted to assemble into rod-like linear arrays (Figure 3, Bottom). These arrays could be cross-linked by any three-arm LMs integrated into the network. Indeed, most biological tissues contain a mixture of two and three LN domain LMs; therefore, the relative abundance of the three LN LMs would determine the level of cross-linking between these LaNt/LM rods and provide a mechanism to fine-tune biomechanical properties. Within this model, excess LaNt α31 would disrupt cross-links and could explain the vessel disruption observed in the LaNt α31 transgenic animals [83].

While LaNt proteins are likely to be capable of influencing network assembly, they could be involved in direct cell signalling. Cell surface receptor binding sites have been identified in LM α-chain LN domains. Specifically, α-chain LN domains can bind heparan sulfate chains of perlecan [88], and a fragment produced from LMα3B N-terminal proteolytic cleavage supported cell attachment in a α3β1 integrin-dependent manner [89], while an LN domain fragment from LMβ1 has also been implicated in the process of epithelial-mesenchymal transition [90]. However, it is important to note that the relative affinities of LN domains for cell surface receptors are orders of magnitude lower than LM LG domain binding. Whether *any* of these putative signalling pathways are physiologically relevant is yet to robustly be determined but is an important unresolved question. When considering signalling effects, LaNt proteins and netrin-4 are very likely to have indirect effects via their biophysical effects. As BMs sequester growth factors, and as LMs are involved in this process [37,38], one might envisage that modulation of the LM network could modify the bind/release rate of these growth factors. Changing LM network status could also conceivably affect the ways in which the LMs are presented to cells [82]. This, in turn, would influence the clustering or localisation of cell surface receptors and impact upon downstream signalling cascades.

Dissecting the direct vs indirect pathways will not be a trivial task but will shed light on how these relatively new proteins elicit their potent effects and more fundamentally to understand the potentially diverse ways through which LMs can influence cell and tissue behaviour.

## Do the LaNt proteins provide two layers of LM regulation?

The findings to date point toward LaNt proteins being potent mediators of BM function. However, their genetic derivation raises the potential for a second layer of LM regulation, at the transcript level. As the LaNt transcripts are derived from LM genes, they share a promoter with their parent gene. Each time transcription is initiated from that promoter, a proportion of the transcripts will make the LaNt instead of the LM. When the efficiency of splicing or the rate at which the intronic cleavage and polyadenylation events are processed changes, then the relative abundance of the LM and LaNt transcripts will also change. Changing this intron-retention ‘splicing switch' so that more LaNt is made, would effectively decrease protein production of the ‘full-length' LM and simultaneously, by increasing LaNt production, produce a protein that disrupts LM networks.

**Figure 5.**
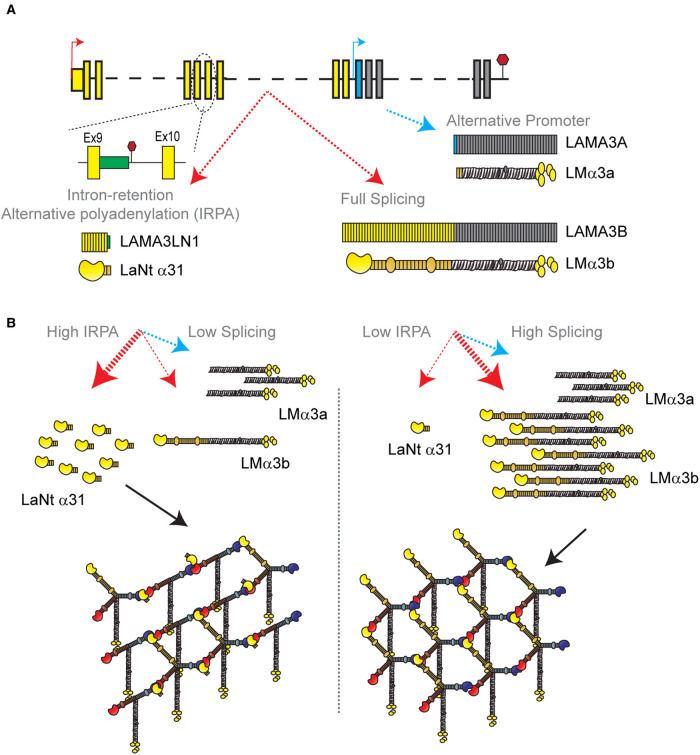
Intron-retention and alternative polyadenylation as a two-layer laminin regulation mechanism. (**A**) Schematic of LAMA3 gene indicating three distinct transcripts and proteins produced. Red arrow — LAMA3B promoter, blue arrow — LAMA3A promoter. Yellow boxes — LAMA3B exons, grey boxes — LAMA3A and B shared exons, blue box — LAMA3A specific exon, green box — protein coding intronic sequence included in LAMA3LN1 but not present in LAMA3B transcript. (**B**) Potential effects of changing the intron-retention/splicing rates on protein production and laminin network assembly.

This concept of dual-layer intron-retention control becomes particularly intriguing when one considers the LAMA3 gene. LAMA3 has two distinct promoters; one which produces the ‘full-length' LMα3b and the LaNt α31 encoding transcripts, and a second promoter that produces the N-terminally truncated LMα3a-chain [76,91] (Figure 5A). Although these two promoters are independently regulated [92], the evolution of the splicing switch allows for the reduction in the ‘full-length' LMα3b by a secondary means (Figure 5B). As LMα3b contains a LN domain and LMα3a does not, reducing the relative production rate of LMα3b would influence the proportion of network-forming LMs being deposited by cells and the mechanical properties of the network. This might be particularly important in tumour situations where the ratio of the LAMA3A to LAMA3B transcripts has been shown to be a better predictor of patient outcome in head and neck squamous cell carcinoma than the expression of either transcript alone [93]. Splicing and polyadenylation rates are frequently dysregulated in cancers but also change in numerous normal situations, including during development and tissue morphogenesis. Determining the contribution of alternative splicing to regulating LM and LaNt production during these processes and their influences on LM networks and biomechanics represents an exciting new area for BM research.

## Conclusions

The central importance of LMs to normal and disease processes has long been established. The LaNt proteins not only expand the LM superfamily but opens up new ways in which LMs are regulated at both the transcript and protein level; they suggest hitherto unknown methods of defining and remodelling BM organisation, biomechanics and signalling, and thereby influencing cell and tissue function in normal and disease situations.

## Perspectives

- The LM family of ECM proteins are essential for normal tissue function with dysfunction associated with inherited and acquired diseases. Central to LM function is their ability to form a network or polymer, with that polymer determining matrix biophysical features, signalling and barrier functions.
- LM polymerisation involves interaction between laminin N-terminal domains; with stable networks requiring a ternary interaction between an α, β and γ laminin chain. The LM family has been extended by additional proteins that contain laminin N-terminal domains but which are not LMs. Emerging results indicate β-type netrins and the LaNt proteins can influence LM network assembly with implications for development and tumour progression.
- The new LaNt proteins and netrin findings generate scenarios where localised and contextually regulated modification of LM networks and BM can occur. Questions remain about the regulation of these modifications and their mechanisms, particularly for situations where LMs are not known to polymerise.

## Open Access

Open access for this article was enabled by the participation of the University of Liverpool in an all-inclusive Read & Publish agreement with Portland Press and the Biochemical Society under a transformative agreement with JISC.

## Abbreviations

BM: basement membrane
DCC: deleted in colorectal cancer
ECM: extracellular matrix
LaNt: laminin N-terminus
LCC, laminin coiled coil; LE repeats: laminin-type epidermal growth factor-like repeats
LM: laminin
LN: laminin N-terminal
STORM: stochastic optical reconstruction microscopy
